# Strain‐Enhanced Ultrastretchable Semiconducting Polymer Fibers for Organic Electrochemical Transistors

**DOI:** 10.1002/advs.78666

**Published:** 2026-11-05

**Authors:** Zixu Chang, Ziao Xu, Qi Tang, Mingjuan Du, Faqiang Wang, Jiexun Zhu, Jianyong Yu, Bin Ding, Zhaoling Li

**Affiliations:** ^1^ Shanghai Frontiers Science Center of Advanced Textiles College of Textiles Donghua University Shanghai China; ^2^ Innovation Center for Textile Science and Technology Donghua University Shanghai China; ^3^ Jiangsu Laboratory of Advanced Functional Materials School of Materials Engineering Suzhou University of Technology Suzhou China

**Keywords:** high stretchability, organic electrochemical transistors, semiconductor polymer fibers, strain‐enhanced electrical performance

## Abstract

Wearable electronics and soft bioelectronics require stretchable devices with steady performance under deformation. However, the balance between electrical performance and stretchability remains a critical obstacle, and most approaches sacrifice charge transport mobility to achieve stretchability. Herein, we present a material design concept that utilizes a wet‐spinning strategy to fabricate highly stretchable semiconducting polymer fibers, enabling high‐performance organic electrochemical transistors (OECTs) with stable or even enhanced charge transport mobility under large strains. The polymer chains are ordered within the fibers under the shear effects of the spinning fluid and can reorient during stretching to dissipate strain energy, further forming a more compact and ordered molecular arrangement. As a result, the fabricated semiconductor fiber can be stretched to over 250% strain and exhibit an ultrahigh normalized transconductance of 276.4 S cm^−1^, far exceeding thin‐film polymer semiconductors. More importantly, the fiber OECTs possess unique strain‐enhanced electrical performance, with substantially improved charge carrier mobility after strain‐induced ordered arrangement of molecular chains. The skin‐like fully stretchable OECTs have been successfully applied for recording electrocardiogram signals. This work provides more possibilities for the further development of stretchable semiconducting polymer materials with high electrical performance.

## Graphical abstract

The continuous fabrication of stretchable polymer semiconductor p(g2T‐T) fibers is realized via wet‐spinning. The p(g2T‐T) semiconductor fibers possess an efficient strain energy dissipation mechanism and unique strain‐enhanced electrical performance. They can be stretched to over 250% strain and exhibit an ultrahigh normalized transconductance of 276.4 S cm^−1^ when used in stretchable organic electrochemical transistors, far exceeding thin‐film polymer semiconductors.

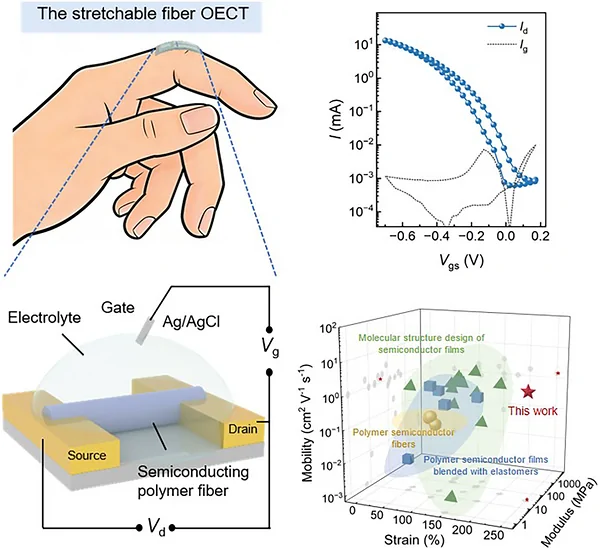

## 1 Introduction

OECTs with mixed ionic‐electronic transport characteristics are highly desirable for developing skin‐like wearable electronics and soft bioelectronics [1, 2, 3, 4, 5]. They can realize the redox reaction in the bulk phase through the ion‐permeable semiconductor channel, allowing them to maintain rapid response times even with longer channel lengths, significantly enhancing signal amplification capabilities [6, 7]. The implementation of OECT sensing function relies on the seamless interface between semiconductor channels and biological tissue surfaces to maintain wearing comfort and high data fidelity in dynamic changes [8, 9, 10]. Thus, inherent stretchability is of paramount importance. Currently, the development of stretchable OECTs has primarily focused on material design at the molecular level of stretchable conjugated polymers and structural engineering of their geometries [11]. These strategies include chemical structural designs that introduce soft segments into the main chains or side chains of semiconductor conjugated polymers [12], the incorporation of multi‐component systems to enhance chain dynamics and increase polymer chain stretchability [13], and the design of material geometries through strain engineering and packaging engineering [14]. However, these strategies typically involve complex synthesis steps and high costs. In addition, these conjugated polymers for OECT are generally based on spin‐coated semiconductor films. The disordered and isotropic morphology of these films may hinder carrier transport, and they poorly maintain performance during stretching [15, 16].

The emergence of fiber electronics has sparked widespread interest among researchers in bioelectronic based on fiber materials [17, 18, 19, 20]. These devices offer advantages such as flexibility, multifunctional integration, and strong adaptability to biological interfaces. Although a few studies have developed semiconducting polymer fibers, they usually lack stretchability, which hinders their application at highly deformable bio‐tissue interfaces when assembled into OECTs [21, 22, 23, 24]. In contrast, stretchable semiconductor polymers with moderate chain flexibility and high OECT performance have attracted widespread attention [25, 26]. However, these are typically thin‐film structures that require complex molecular design to achieve a trade‐off between stretchability and electrical performance. Developing them into fiber structures could potentially simplify this challenge and open up broader application possibilities. Therefore, there is a dearth of development of stretchable polymer semiconductor fibers, not to mention understanding the mechanism of high stretchability and the relationship between morphological evolution and charge carrier transport during stretching.

To address the above challenges, we developed a nondoped redox‐active semiconductor polymer named poly[3,3'‐bis[2‐[2‐(2‐methoxyethoxy)ethoxy]ethoxy]‐2,2':5',2''‐terthiophene‐5,5''‐diyl] (p(g2T‐T) with a continuous fiber structure using wet spinning (Figure 1a). Obtained p(g2T‐T) fibers with excellent anisotropy that enable the molecular chains to distribute stress along the axial direction and undergo efficient chain slippage during stretching. The p(g2T‐T) fibers present high stretchability and maintain superior OECT performance even under a strain of 250%, which has not been observed before. Stretched p(g2T‐T) fibers exhibit favorable molecular packing, demonstrating a higher normalized transconductance than OECTs based on p(g2T‐T) films, reaching up to 276.4 S cm^−1^. Excitingly, p(g2T‐T) fibers exhibit unique strain‐enhanced electrical performance. The stretched fibers feature a smaller *π*–*π* stacking distance and enhanced polymer chain alignment, thereby increasing hole mobility. Furthermore, we demonstrate p(g2T‐T) fiber‐based fully stretchable OECT for biosensing at the skin interface (Figure 1b). This OECT exhibits excellent amplification capability and responsiveness in electrocardiogram (ECG) monitoring, providing a promising strategy for the development of bioelectronic devices for personalized health monitoring.

**FIGURE 1:**
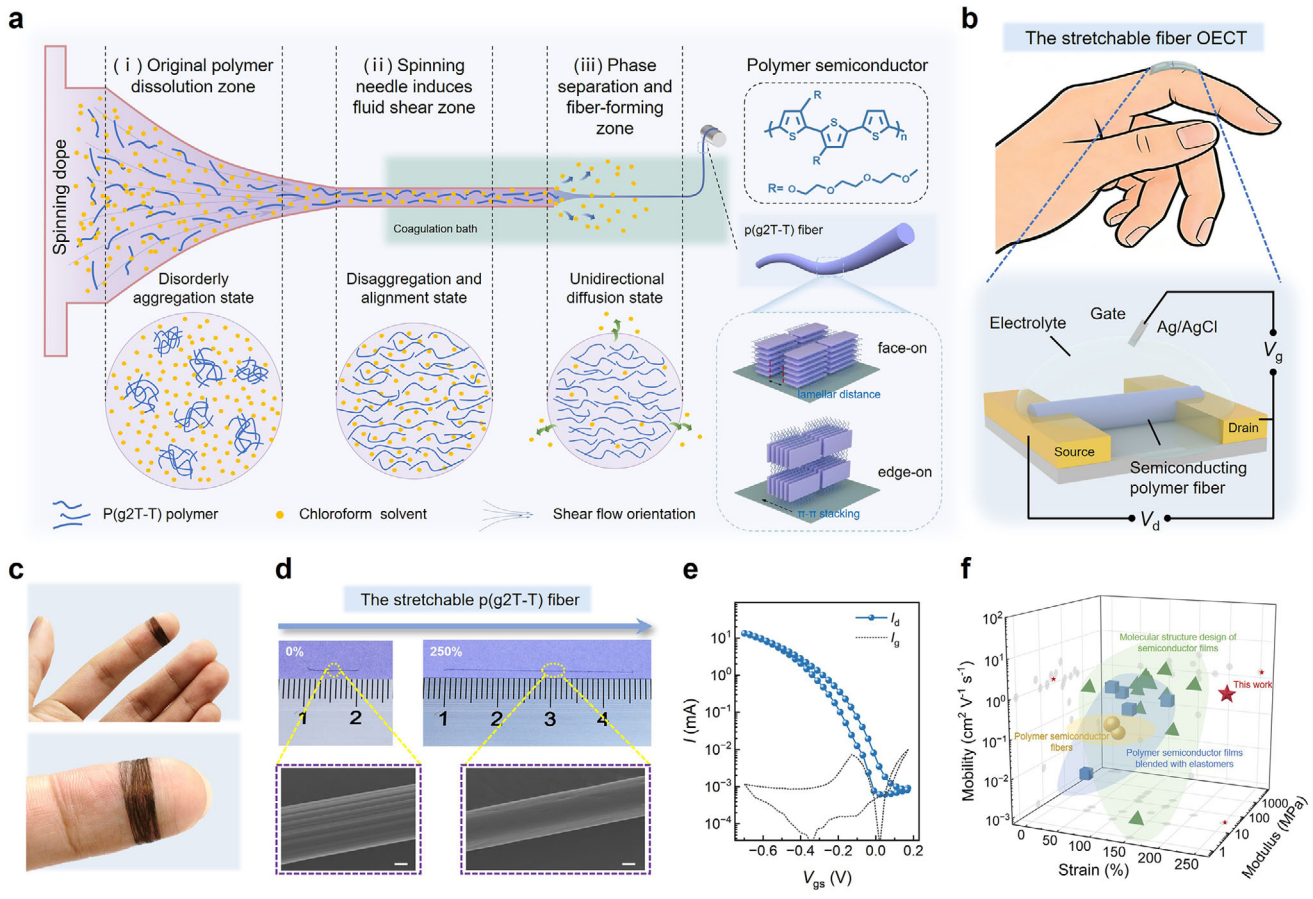
The fabrication and applications of ultrastretchable p(g2T‐T) fibers. (a) Schematic diagram of the wet spinning process for fabricating p(g2T‐T) fibers. (b) Schematic illustration of stretchable OECTs for skin‐interface sensing based on p(g2T‐T) fibers. (c) Photograph showing the prepared p(g2T‐T) fiber conforming to the skin. (d) Optical images of p(g2T‐T) fiber stretched to 250% and SEM images (scale bar, 5 µm) showing the initial and stretched states. (e) p(g2T‐T) fiber serving as the channel layer in an OECT exhibits normal transport behavior. (f) Comparison of the elongation at break, Young's modulus, and charge carrier mobility of p(g2T‐T) fibers with other reported stretchable semiconductors [12, 13, 14, 24, 25, 31, 32, 33, 34, 35, 36, 37, 38, 39, 40, 41, 42, 43, 44, 45, 46].

## 2 Results and Discussion

### 2.1 Fabrication of p(g2T‐T) Semiconductor Fibers

The semiconductor polymer p(g2T‐T) used in this study is a p‐type polymer composed of a polythiophene main chain and triethylene‐glycol side chains. It has a high molecular weight (Table S1) and an electron‐rich conjugated system. With moderate main chain planarity, nonlinear backbone of moderate flexibility, and polar side chains, the p(g2T‐T) exhibits excellent charge delocalization and ion transport properties [27]. Unlike conventional high‐concentration wet spinning, we successfully achieved continuous production of p(g2T‐T) fibers by reducing the concentration of the semiconducting polymer and using a custom‐designed wet spinning equipment (Figure 1a,c). P(g2T‐T) fibers exhibit outstanding spinnability and can be formed into fibers within a concentration range of 5–15 mg mL^−1^ using various needle sizes and coagulation baths (Figure S1).

Since the quality of wet‐spinning fibers depends largely on the spinning solution formulation and the choice of a suitable coagulation bath, we determined the optimal wet‐spinning conditions (15 mg mL^−1^, 0.9 mL h^−1^, ethanol coagulation bath) by optimizing the spinning solution concentration (Figure S2), shear speed (Figure S3), and type of coagulation bath (Figure S4), thereby producing p(g2T‐T) fibers with high stretchability and favorable electrical performance. In addition to the inherent properties of the p(g2T‐T) polymer, the high stretchability of the fibers is primarily attributed to the fiber formation process during wet spinning, which mainly involves solution shearing and phase‐separation solidification. After dissolution, the polymer undergoes intense disordered aggregation in the solution, resulting in a highly entangled and irregularly agglomerated state with large aggregate sizes. The use of a long, narrow needle and an appropriate shear speed can reduce aggregate size and align them along the shear direction (Figure 1a and Figure S5). The phase‐separation process involves solvent diffusion and the solidification of the polymer into fibers after the solution flows into the coagulation bath. However, this process is relatively complex and is influenced by solubility, surface energy, and the crystallization and aggregation kinetics [28]. To achieve superior charge transport and stretchability, we used Hansen solubility parameter theory to guide the solvent diffusion process [29, 30]. Table S2 lists the solubility parameters for chloroform and several coagulation baths, and compares the contributions of each component and the energy difference (Δδ) between them (Figure S6). When Δδ is close between two solvents, the solvent diffusion mode is rapid bidirectional diffusion. Conversely, a large Δδ leads to slow unidirectional diffusion, which typically favors slow phase transitions, crystallization, and relatively low orientation in the fibers, thereby providing greater stretching freedom. Compared to films formed by rapidly drying polymer solutions after spin‐coating (which typically tend to create stress concentrations and microscopic defects within the material), wet‐spun fibers offer greater flexibility for reorientation and alignment to dissipate strain energy, ultimately resulting in a tensile elongation of up to 250% (Figure 1d and Movie S1). This is because molecular chains that are moderately aligned along the axial direction can effectively transmit and disperse stress along the chain when subjected to axial stress. When used as channels for OECT, the fibers also exhibit ideal OECT performance (Figure 1e). Compared to other stretchable polymer semiconductor films/fibers, this p(g2T‐T) fiber achieves unprecedented stretchability and a Young's modulus matching biological tissues, while maintaining a high carrier mobility (Figure 1f and Table S3).

### 2.2 Morphological, Electrical, and Mechanical Properties of High‐Stretched p(g2T‐T) Fibers

To elucidate the reasons for the retention of superior stretchability and charge transport properties in the fibers, we performed morphological characterization to reveal the structure at both macroscopic and microscopic scales. At the macroscale, polarized optical microscopy (POM) was used to analyze the long‐range order of polymer molecular chains in the films and fibers. POM revealed that p(g2T‐T) fibers transitioned from total extinction to the reappearance of light at 45°, indicating that the polymer main chains achieved long‐range order along the fiber axis (Figure 2a). However, no significant optical anisotropy is observed in p(g2T‐T) films. Furthermore, after stretching the fibers, the 45° light becomes more pronounced and is distributed throughout the fiber, which also indicates that stretching promotes chain alignment (Figure S7). At the microscale, ultraviolet‐visible (UV‐vis) absorption spectra were used to characterize the degree of short‐range ordering and the conformation of polymer chains in films and fibers. Compared to p(g2T‐T) solutions and films, p(g2T‐T) fibers show a significant red shift of the 0‐0 peak (Figure 2b), indicating an increase in short‐range ordering. Photoluminescence (PL) spectra also confirm the short‐range order in the fibers (Figure S8). Combining UV absorption and PL spectra, we propose that polymers in the one‐dimensionally confined fibers may adopt a more planar main‐chain conformation compared to those in films. This improvement in morphological arrangement and conformation may lead to significantly enhanced electrical performance in the fibers. To verify this hypothesis, we extracted the optical bandgap energies of the fibers and films from their UV–vis spectra. In comparison with the films, the p(g2T‐T) fibers showed a lower bandgap energy (Figure 2c), indicating better π‐electron delocalization. This can be explained by the increased short‐range clustering resulting from the flatter chain conformation in the fibers [29].

**FIGURE 2:**
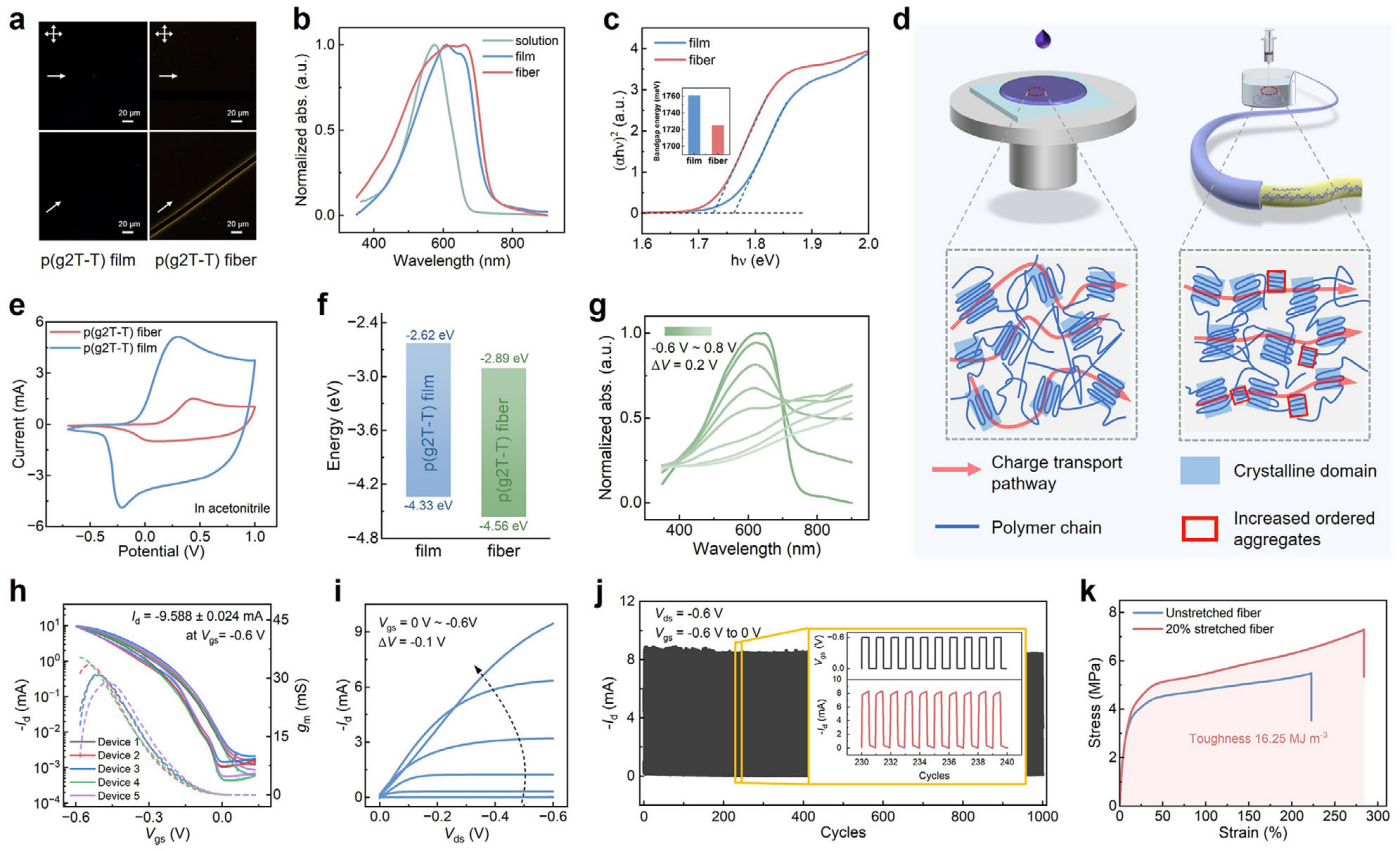
Morphological, electrical, and mechanical properties of p(g2T‐T) fibers. (a) Polarized optical microscopy images of p(g2T‐T) fibers and films. (b) Comparison of UV absorption spectra of the p(g2T‐T) solution, spin‐coated film, and wet‐spun fiber. (c) Normalized UV absorption of p(g2T‐T) films and fibers. The inset shows their bandgap energies. (d) Schematic illustration of charge transport mechanisms in materials (spin‐coated films and wet‐spun fibers) under different fabrication strategies. (e) CV measurements of p(g2T‐T) films and fibers. (f) Energy level diagram of p(g2T‐T) polymers. (g) Operando UV‐vis measurements on a p(g2T‐T) fiber. (h) Transfer and transconductance curves of five different fiber OECTs at *V*_ds_ = −0.6 V. (i) Output curve of fiber OECT. (j) Operational stability of fiber OECT under 1000 on‐off cycling measurements with square‐wave gate bias. The inset shows a zoomed‐in view from 230 to 240 cycle. (k) Stress‐strain curves of unstretched and 20% stretched fibers.

Furthermore, we investigated the electrochemical behavior of p(g2T‐T) fibers to understand their electronic structure. Cyclic voltammetry (CV) (Figure 2e and Figure S9) and UV–vis absorption spectra were employed to evaluate the highest occupied molecular orbital (HOMO) and lowest unoccupied molecular orbital (LUMO) energy levels of p(g2T‐T) films and fibers. The calculation methods and results are summarized in Table S4. The p(g2T‐T) fiber exhibits lower LUMO/HOMO energy levels compared to the film (Figure 2f), which helps promote electron generation and enhances operational stability in aqueous solutions [47]. We characterized the electrochemical stability in two types of electrolytes: 0.1 M NaCl solution and 0.1 M deoxygenated ultra‐dry acetonitrile solution containing tetrabutyl‐ammonium hexafluorophosphate as the electrolyte. Both the fibers and the films exhibit promising electrochemical stability (Figure S10), which contributes to the operational stability of the fabricated OECT devices [48]. The redox activity and ionic doping characteristics of the fibers were further investigated via spectroelectrochemistry. Similar to the films, the fibers display electrochromic property, and their absorption spectra exhibit analogous changes (Figure 2g and Figure S11). Based on the above analysis, we infer that the molecular chain arrangement of the stretchable semiconductor fibers prepared by wet spinning may be more conducive to charge transport compared to spin‐coating films (Figure 2d) [49].

Unstrained fibers serve as channel materials for OECTs. The p(g2T‐T) fiber OECT exhibits typical p‐type accumulation mode behavior, and the performance of the OECT is significantly enhanced (Figure 2h,i). The normalized transconductance (*g*_m normalized_) of the unstretched fiber OECT reaches as high as 83.2 S cm^−1^. To compare intrinsic OECT performance, we calculated the quality factor *µC* ^*^ of the fiber OECT, reaching 207.24 F cm^−1^ V^−1^ s^−1^. This value is more than twice that of the p(g2T‐T) film OECT (Figure S12). This improvement in transistor performance can be attributed to the increased carrier mobility resulting from the more ordered arrangement of molecular chains within the fiber (Figure 2d and Figure S13). Furthermore, the current of the p(g2T‐T) fiber OECT shows almost no decay after 1000 on‐off cycling measurements under pulsed gate voltage (Figure 2j). The on‐off cycling stability of this device corresponds to the excellent electrochemical stability of the fiber in aqueous electrolytes.

The mechanical properties of the fibers are critical for the fabrication of stretchable devices and biosensing applications at the skin interface [50, 51, 52]. We performed cyclic stretch‐recovery test at 20% strain on untreated fibers, demonstrating that they can undergo plastic deformation (Figure S14). We further investigated the effect of post‐stretching treatment on the mechanical properties of p(g2T‐T) fibers. Figure 2k shows a typical stress‐strain curve of p(g2T‐T) fibers, which exhibit moderate mechanical strength and ultrahigh stretchability. Excitingly, fibers subjected to a 20% stretching treatment during the spinning process exhibit superior mechanical properties (Figure S15), featuring ultrahigh strain at break (> 250%), excellent toughness (> 15 MJ m^−3^), and a Young's modulus (32.4 ± 0.05 MPa) that matches biological tissues (skin, cartilage, and nerves). This indirectly demonstrates that stretching promotes the alignment of molecular chains within the fibers and enhances the electrical performance of the devices, which will be verified in subsequent investigations. Overall, these characteristics make them highly suitable for wearable/implantable stretchable bioelectronic.

### 2.3 Strain‐Enhanced Electrical Performance of p(g2T‐T) Fibers

To investigate the effect of strain on the p(g2T‐T) fiber OECTs performance, we assembled typical top‐gate OECT devices by stretching the fibers to different lengths (0%–250%) (Figure 3a). After stretching along the fiber axis, the transfer curve (Figure 3b) and output curve (Figure S16) of the OECT remained virtually unaffected, with only a slight decrease in the peak *g*_m_ from 44.7 to 36.8 mS. This is primarily attributed to the reduction in the thickness and width of the OECT channel caused by the reduction in fiber diameter after stretching. To more accurately characterize the effect of strain on the intrinsic performance of the OECT, changes in device dimensions must be taken into account. Considering the reduced fiber diameter after stretching, we evaluated the change in normalized *g*_m_. Notably, as strain increases, the *g*_m, normalized_ of the p(g2T‐T) fiber OECT rises significantly, from an initial value of 103.6–276.4 S cm^−1^ at 250% strain (Figure 3c). The trend of the quality factor µC^*^ is consistent with that of *g*_m, normalized_, reaching a maximum of 484.9 F cm^−1^ V^−1^ s^−1^ (Figure 3c). We calculated *V*_th_ under different strains using the transfer curve (Figure S17), and the results indicate that the change in *V*_th_ is less than 0.07 V (Figure 3d). This is corroborated by the CV results of the fiber under different strains (Figure S18), indicating that the oxidation onset voltage remains virtually unchanged. In addition, we measured the volume capacitance C^*^ of the fiber using electrochemical impedance spectroscopy (Figure S19). The change in C^*^ during stretching is less than 5% (Figure 3d). Based on this, we calculated the carrier mobility under different strxaioyuains and found that *µ* increased from 0.78 to 1.41 cm^2^ V^−1^ s^−1^, consistent with the changes in *g*_m, normalized_ and µC^*^ (Figure 3c). We evaluated the transient behavior of fiber OECT under different strains. The on‐off time after stretching is slightly reduced, primarily because the fibers become thinner, resulting in a decrease in channel thickness (Figure S20). This short response time can meet the requirements of most biosensing applications. From the above results, the unique strain‐enhanced electrical performance is primarily attributed to the increase in *µ* of the p(g2T‐T) fibers induced by stretching. At the molecular structural level, we infer that strain optimizes the conformation and arrangement of molecular chains within the fibers [25]. Additionally, we evaluated the robustness of the p(g2T‐T) fiber OECT under stretching cycles. After 1000 stretching cycles, the OECT performance suffered only minimal degradation (Figure S21), ensuring its reliability for repeated use.

**FIGURE 3:**
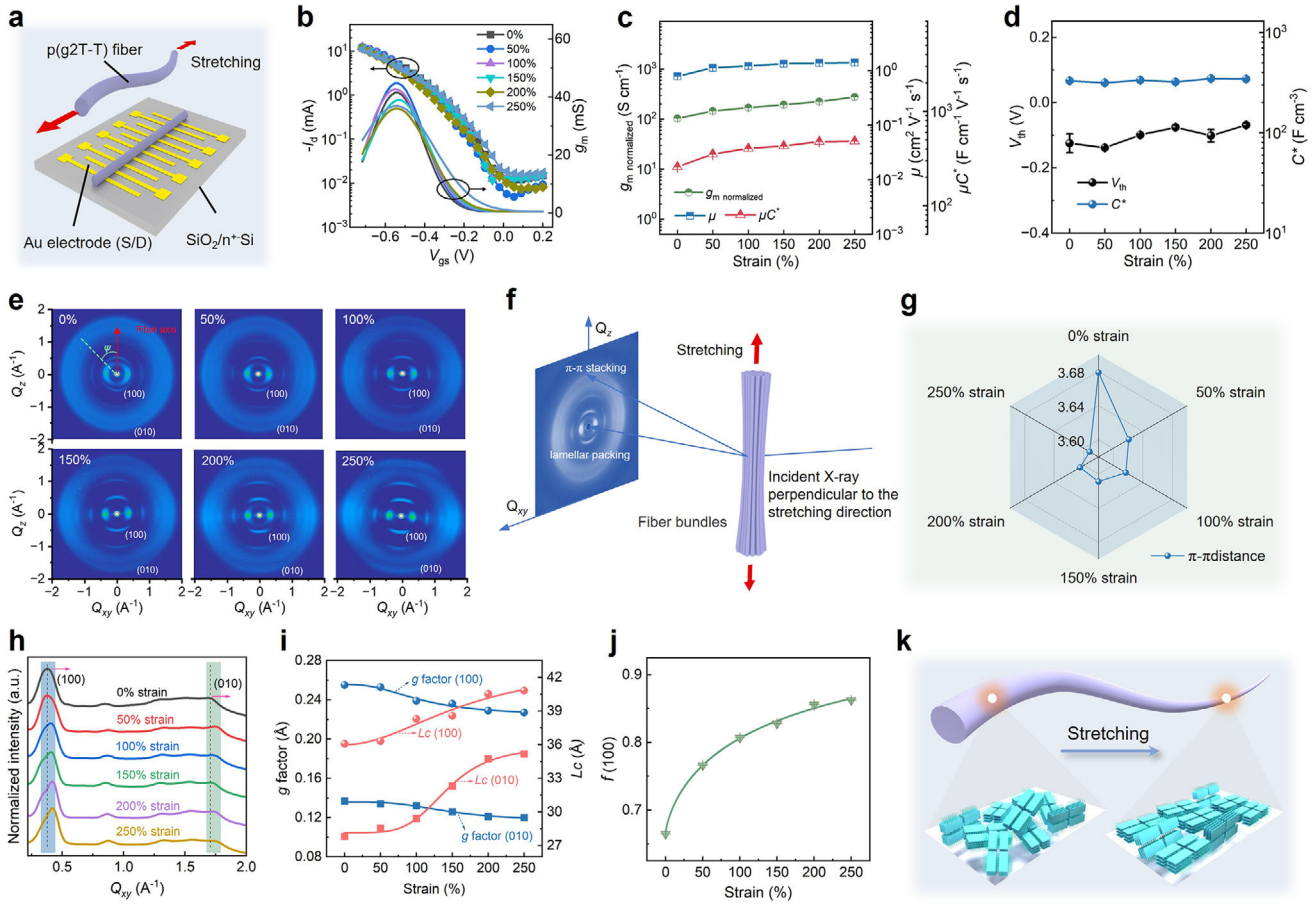
Characterization of the electrical performance of p(g2T‐T) fibers under different strains and the mechanism of strain‐enhanced electrical performance. (a) Schematic diagram of the OECT structure composed of stretching fibers. (b) Transfer curves and *g*_m_ of the stretched p(g2T‐T) fiber OECT. c *g*_m, normalized_, µC^*^, and *µ* of p(g2T‐T) fiber OECTs under different strains. (d) *V*_th_ and C^*^ of the stretched p(g2T‐T) fiber. (e) The WAXS pattern of the p(g2T‐T) fiber under different strains. (f) Schematic illustration of the stretched fiber WAXS measurement. (g) The *π*–*π* distance of the fiber under different strains. (h) Line cut of the WAXS patterns for fibers under different strains. (i) Comparison of the *g* factor and *L*_C_ of the stretched p(g2T‐T) fiber. (j) Comparison of the *f* of the stretched p(g2T‐T) fiber. (k) Schematic illustration of changes in the crystalline structure of the stretched p(g2T‐T) fiber.

To verify our hypothesis that the molecular packing structure in p(g2T‐T) fibers evolves more favorably under stretching, the fibers at different strains were characterized using wide‐angle X‐ray scattering (WAXS). The scattering pattern indicates that the polymer molecules in the fibers exhibit a mixed packing behavior, comprising both face‐on and edge‐on orientations (Figure 3e). Since the incident X‐rays are perpendicular to the fiber axis, the higher scattering intensity observed indicates that the polymer chains in the p(g2T‐T) fibers are highly aligned along the strain direction (Figure 3e,f). Furthermore, as strain increases, the relative intensity of the (100) lamellar packing peak increases (Figure 3h), which is likely due to large strains along the fiber axis causing the edge‐on molecular packing to rotate into face‐on packing [25, 53]. We find that the lamellar distance of the stretched fiber decreases from 16.7 to 14.8 Å, and the *π*–*π* distance shrinks from 3.68 to 3.59 Å, indicating a more compact arrangement of the p(g2T‐T) fiber molecular chains (Figure 3g and Figure S22). Moreover, we calculated the coherence length (*Lc*) [54] and the para‐crystalline disorder (*g* factor) [55] of the fiber under stretching, which are important parameters describing the degree of order and disorder of the polymer molecular chains during the packing process, respectively. As the strain increased, the *Lc* of the p(g2T‐T) fiber increased significantly, while the *g* factor gradually decreased (Figure 3i). At the same time, we extracted the Herman's orientation factor (ƒ) [56] from the azimuthal intensity map of the (100) peak of the p(g2T‐T) fiber (Figure S23) to quantify the chain orientation of the fiber. As the fiber is stretched from 0% to 250%, the ƒ increases from 0.66 to 0.86 (Figure 3j). All molecular packing parameters of p(g2T‐T) fibers under strain are summarized in Table S5. These results indicate that the molecular chains of the fibers adopt a more compact and ordered arrangement under stretching (Figure 3k), which explains the enhanced OECT performance under strain. Simultaneously, this arrangement of polymer chains along the strain direction and the face‐on rotation along the fiber axis effectively dissipate strain energy, further explaining why p(g2T‐T) fibers can achieve ultrahigh stretchability. This unique strain‐enhanced OECT performance can be understood from the perspective of OECT device physics. In an OECT, ions from the electrolyte are injected into the channel, thereby altering its overall doping state and conductivity. According to the Bernards model, the *g*_m_ is given by Equation (6), where *d*, *µ*, C^*^, and *V*_th_ can all influence performance in principle, but the product µC^*^ defines the material's intrinsic performance metric. After eliminating device‐size effects, the *g*_m, normalized_, is given by Equation (7). It directly reflects the material's intrinsic OECT performance. Although the absolute *g*_m_ decreases slightly under stretching (due to a decrease in *d*), the *g*_m, normalized_ and µC^*^ are significantly enhanced. Notably, C^*^ and *V*_th_ remain essentially unchanged under stretching. Therefore, this enhancement is effectively an enhancement of *µ* and does not come at the expense of ionic storage capacity. It essentially stems from the structural evolution of the material, which is consistent with the WAXS results.

### 2.4 Fully Stretchable p(g2T‐T) Fiber‐Based OECT and Applications at the Skin Interface

Benefiting from the ultrahigh stretchability of p(g2T‐T) fibers, a fully stretchable OECT was fabricated (Figure 4a). The substrate material employs SEBS, which exhibits excellent flexibility, while the electrodes consist of a stretchable conductive silver paste capable of maintaining low sheet resistance under strain and repeated stretching cycles (Figure S24). The obtained OECT device exhibits outstanding robustness, withstanding continuous stretching, twisting, and even poking (Figure 4b). The fully stretchable fiber OECT demonstrates ideal device performance, with a peak *g*_m_ reaching 8.5 mS (Figure 4c and Figure S25). The OECT can still be stretched up to 250%, and the transfer curve (Figure S26) and output curve (Figure S27) show only a slight decrease in conductance current and a minor reduction in *g*_m_ (Figure 4d), which is attributed to changes in the device channel dimensions caused by stretching. Furthermore, this fully stretchable OECT exhibits high durability over 1,000 repeated stretching cycles at 200% strain (Figure 4d and Figure S28).

**FIGURE 4:**
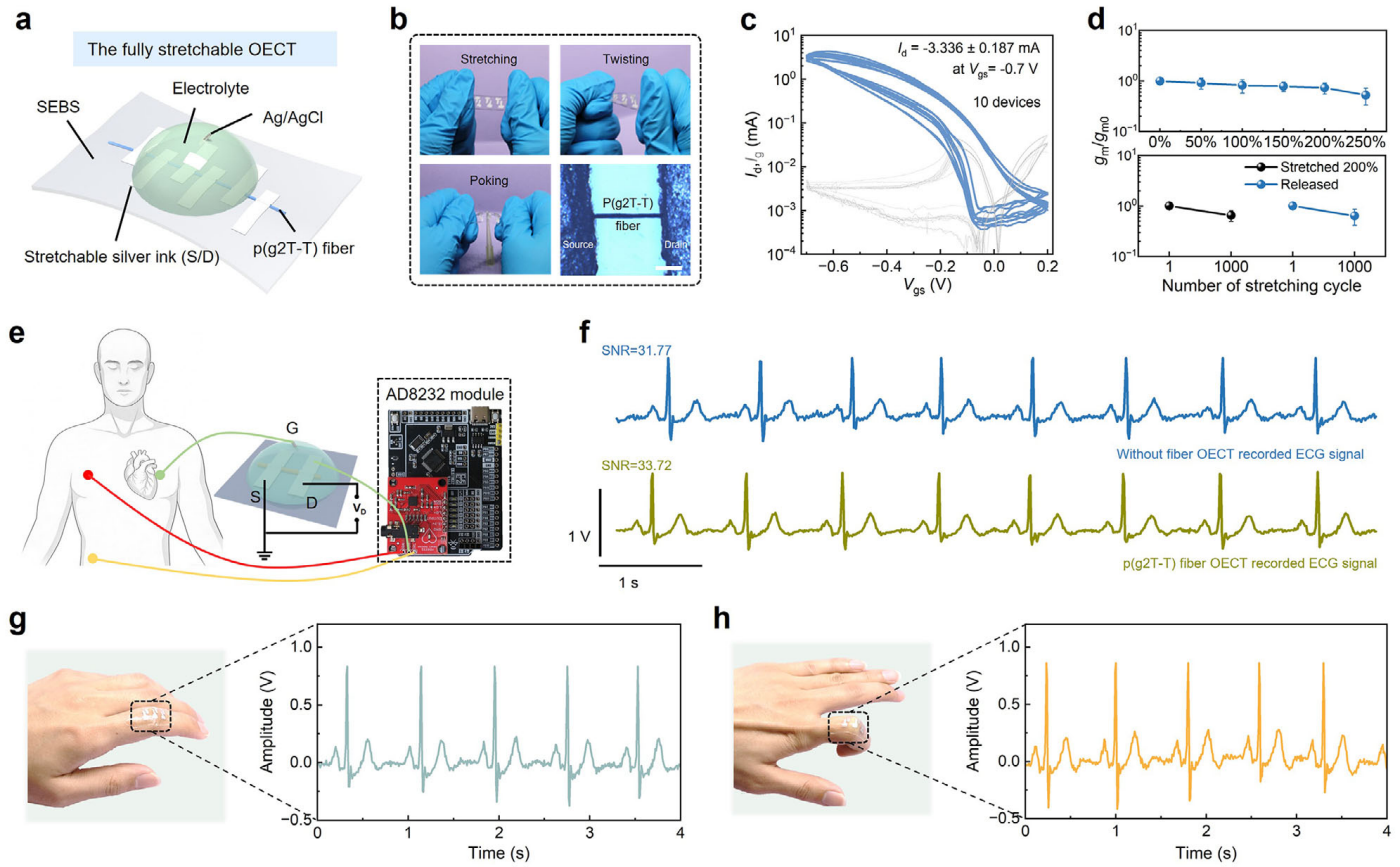
The fully stretchable OECT performance and its applications in ECG signal monitoring. (a) Schematic diagram of the assembly of a fully stretchable OECT. (b) Photographs of fully stretchable OECT under stretching, twisting, and poking with a sharp object and magnified channel optical images (scale bar, 200 µm). (c) Transfer curves of 10 fully stretched fiber OECTs. (d) Changes of *g*_m_ under 0%–250% strain and during stretching cycles. (e) Schematic diagram of the fully stretchable OECT connected to the human body for ECG recording. (f) Comparison of ECG signals acquired using a commercial module vs. those acquired using a fiber OECT as the amplifier. (g) Photograph of finger straight and corresponding ECG signal. (h) Photograph of finger bending and corresponding ECG signal.

We further mount this stretchable OECT, with high transconductance and superior stability, onto human skin as a bioamplifier to record ECG signals. Owing to the inherent conformability of the SEBS substrate, the stretchable OECT maintains close contact with the skin surface [57]. Additionally, transparent tape is used for extra fixation to ensure stable ECG monitoring during dynamic movements. By connecting the gate and source to the human body, the faint ECG signals are amplified into drain current for recording (Figure 4e). To better visualize ECG signals, we utilize stretchable OECT as a preamplifier, while a commercial ECG module (AD8232) performs backend signal processing and conversion [58]. Compared to ECG signals recorded directly by the AD8232, those amplified by our p(g2T‐T) fiber OECT exhibit a higher signal‐to‐noise ratio (SNR), reaching up to 33.72 (Figure 4f). Furthermore, it is crucial to have a stable ECG monitoring capability under mechanical deformation. To fully demonstrate the reliability of our stretchable OECT, we recorded ECG signals at the finger joint, which undergoes a large range of deformation (Figure 4g,h). The results show that the fully stretchable OECT maintains stable ECG signal acquisition even after repeated large deformations (Figure S29). Overall, the p(g2T‐T) fiber stretchable OECT holds great potential for personalized health monitoring applications.

## 3 Conclusions

Stretchable OECTs based on wet‐spun fibers were demonstrated, maintaining stable current output even under strains as high as 250%. The highly efficient strain energy dissipation mechanism primarily stems from the high degree of orientation freedom in fibers produced by wet spinning, allowing molecular chains to reorient along the fiber axis during stretching. Concurrently, stretching significantly improves *π*–*π* and lamellar packing, enhancing molecular chains compactness and order, which endows stretchable OECTs with unique strain‐enhanced electrical performances. By integrating ultra‐stretchable fibers with other components, fully stretchable OECTs have been successfully developed, enabling the recording of ECG signals with high SNR on human skin. Unlike previous studies on stretchable OECTs primarily focused on film devices, this study shows that fibers produced via the spinning method can simultaneously and significantly enhance both the stretchability and electrical properties of semiconducting polymers. Therefore, this processing approach is expected to serve as a universal strategy for fabricating stretchable semiconducting polymers in addition to molecular engineering design, thereby promoting the development of high‐performance, mass‐produced stretchable bioelectronics.

## 4 Experimental Section

### 4.1 Materials

Polymer semiconductor p(g2T‐T) was purchased from Derthon Optoelectronic Materials Sci. Tech. Co., Ltd. Chloroform was purchased from Sigma‐Aldrich. Anhydrous ethanol, isopropanol, tetrahydrofuran, acetone, n‐butyl acetate, toluene, and methanol were all purchased from Sinopharm Chemical Reagent Co., Ltd. Acetonitrile, tetrabutyl‐ammonium hexafluorophosphate, ferrocene, and sodium chloride were purchased from MACKLIN. Stretchable conductive silver paste was purchased from Kezhizhu Electronics Technology Chongqing Co., Ltd. SEBS H1062 was purchased from Asahi Kasei.

### 4.2 Fabrication of p(g2T‐T) Fibers

First, the p(g2T‐T) polymer (5, 10, 15 mg) was dissolved in 1 mL of chloroform at 60°C for 8 h to prepare the spinning solution. Then, the p(g2T‐T) solution was loaded into syringes fitted with needles of different diameters and lengths. Next, using a syringe pump (LSP01) at room temperature, the spinning solution was extruded from the syringes at different rates (0.3, 0.6, 0.9, 1.2 mL h^−1^) under needle shear into various coagulation baths for phase separation and fiber formation. Finally, the fibers were continuously collected on a roll for drying.

### 4.3 Morphology Characterization

The microscopic morphology of the fibers was analyzed using scanning electron microscope (VEGA3 LUM, Czech Republic). POM images were obtained using a polarizing microscope (BA310POL, Motic China Group Co., Ltd., China). Absorption spectra were recorded on an UV‐Vis‐NIR spectrometer (UV3600, Shimadzu, Japan). PL spectra were measured via a steady‐state/transient fluorescence spectrometer (FL‐QM, China). Dynamic light scattering (DLS) measurements were recorded using a dynamic/static laser light scattering instrument (BI‐200SM, USA).

WAXS experiments were conducted on a Xeuss 3.0 (Xenocs, France). The signal source was a Cu target at 8.05 keV, with a wavelength of 1.54189 Å. Data processing was performed using the Foxtrot software to obtain intensity integrals over q and the azimuthal angle ψ. The para‐crystalline disorder (*g* factor), coherence length (*Lc*), and Herman's orientation factor (ƒ) were calculated based on the (100) or (010) peaks, which according to the following equations:

(1) $$g=\sqrt{\frac{\Delta q}{2\pi q_{0}}}\;$$

(2) $$L_{c}=0.89\times \frac{2\pi }{\Delta q}$$

(3) $$f=\frac{180-H}{180}$$

where *Δq* is the full width at half maximum (FWHM) of the diffraction peak, *q_0_* is the center position of the diffraction peak, and H is the FWHM of the azimuthal intensity pattern corresponding to the (100) peak.

### 4.4 Mechanical Performance Characterization

The p(g2T‐T) fiber was first adhered to a rigid card with a 1 cm aperture using adhesive tape. The sample was then placed in the clamp fixture of the single fiber strength tester (ST200C, Suzhou Rising Intelligent Technology Co., Ltd., China). Finally, the rigid card was cut to initiate the test (at a tensile speed of 5 mm/min) to record the stress‐strain curve. The fiber's toughness and Young's modulus were calculated from these data.

### 4.5 Electrochemical Characterization

Electrochemical experiments were performed on an electrochemical workstation (CHI660, China). p(g2T‐T) film/fiber transferred onto indium tin oxide conductive glass served as the working electrode, Pt wire was used as the counter electrode, and Ag/AgCl was used as the reference electrode. Use a 0.1 M NaCl aqueous solution or a 0.1 M tetrabutyl‐ammonium hexafluorophosphate solution in deoxygenated, super‐dry acetonitrile as the electrolyte. For CV measurements, the test was conducted in the range of −0.7 to 1 V at a scan rate of 0.1 V/s. The HOMO and LUMO energy levels were calculated based on the CV test results, and the calculation methods and results are detailed in Table S4. For EIS measurements, an AC offset of 5 mV (RMS amplitude) and a DC voltage bias of 0.6 V were applied, recording EIS data from 10 kHz to 0.1 Hz. EIS data analysis was performed using the ZSimpWin. The effective capacitance *C* and volume capacitance C^*^ were calculated according to the following equations:

(4) $$C=\frac{1}{2\pi fIm\left(Z\right)}$$

(5) $$C^{*}=\frac{C}{d\times A}\;$$

where *f* is the frequency, *Im(Z)* is the imaginary part *Z''*, *d* is the sample thickness, and *A* is the electrode area.

Spectroelectrochemistry measurements were performed on the Shimadzu UV‐3600 UV‐Vis‐NIR spectrophotometer and the CHI660E workstation. A three‐electrode system was used in a quartz cuvette, and the background absorbance of the cuvette/electrolyte/ITO was removed using a blank ITO slide prior to sample measurement. Before recording the spectra, an initial bias of 5s was applied to the sample (to ensure the sample was at the non‐doped state). During the scan, the working voltage was stepped from −0.6 to 0.8 V in 0.2 V increments, and the absorption spectra of the sample were recorded at each bias voltage.

### 4.6 Fiber OECTs and Characterization

SiO_2_ wafers or transparent glass are used as substrates after treatment. Subsequently, fibers are placed on the substrate, and source and drain electrodes (Au/Ag paste) are deposited using a mask. The 0.1 M NaCl solution is applied as the electrolyte, and Ag/AgCl electrodes (Warner Instruments) are used as the OECT gate electrodes. The channel length (*L*) is 100 µm, while the channel width (*W*) and equivalent thickness (*d*) depend on the fiber diameter. The fibers present an elliptical cross‐sectional shape with uneven thickness. Accordingly, we convert the fiber thickness (*d_0_*) to the equivalent channel thickness. Two Keithley 2450 semiconductor parameter analyzers were used to record the transfer and output curves of the OECT devices. Device stability measurements were obtained by applying continuous gate pulse voltages using a FeelElec FY6900 function signal generator. The *g*_m, normalized_, µC^*^, *µ*, and *d* of the fiber OECT were calculated according to the following equations:

(6) $$g_{m}=\frac{\partial I_{d}}{\partial V_{gs}}=\frac{Wd}{L}\;\mu C^{*}\left(V_{th}-V_{gs}\right)$$

(7) $$g_{m,\;normalized}=\frac{g_{m}\times L}{Wd}$$

(8) $$d=\frac{\frac{1}{4}\pi Wd_{0}}{W}=\frac{1}{4}\;\pi d_{0}$$

where *W*, *d*, *d_0_*, and *L* represent the channel width, equivalent thickness, fiber thickness, and length, respectively; *V_th_* is the threshold voltage, and *V_gs_* is the applied gate voltage.

### 4.7 ECG Signal Recording

The stretchable OECT used SEBS as the substrate and stretchable conductive silver paste as the source and drain electrodes. The assembly of other components follows the method described above. For ECG signal recording, a three‐electrode configuration using commercial gel electrodes was employed, with the electrodes positioned beneath the left clavicle, beneath the right clavicle, and on the right thigh. The electrode beneath the left clavicle was connected to the gate electrode of the OECT, and the ECG signal was recorded using an AD8232 and a microprocessor.

## Author Contributions

**Zixu Chang**: validation, investigation, project administration, visualization, writing – original draft. **Ziao Xu**: visualization, validation. **Qi Tang**: project administration, formal analysis. **Mingjuan Du**: formal analysis, project administration. **Faqiang Wang**: visualization, investigation. **Jiexun Zhu**: investigation, validation. **Jianyong Yu**: writing – review and editing, conceptualization, supervision. **Bin Ding**: writing – review and editing, conceptualization, resources. **Zhaoling Li**: conceptualization, methodology, writing – review and editing, funding acquisition.

## Conflicts of Interest

The authors declare no conflicts of interest.

## Supporting information




**Supporting File 1**: advs78666‐sup‐0001‐SuppMat.docx.

**Supporting File 2**: advs78666‐sup‐0001‐MovieS1.MOV.

## Data Availability

The authors declare that the data supporting the findings of this study are available within the paper and its supplementary information files, and all data are available from the corresponding author upon request.

## References

[1] D. Liu , X. Tian , J. Bai , et al., “A Wearable In‐Sensor Computing Platform Based on Stretchable Organic Electrochemical Transistors,” Nature Electronics 7, no. 12 (2024): 1176–1185, 10.1038/s41928-024-01250-9.

[2] Y. Wang , S. Wustoni , J. Surgailis , Y. Zhong , A. Koklu , and S. Inal , “Designing Organic Mixed Conductors for Electrochemical Transistor Applications,” Nature Reviews Materials 9, no. 4 (2024): 249–265, 10.1038/s41578-024-00652-7.

[3] N. A. Kukhta , A. Marks , and C. K. Luscombe , “Molecular Design Strategies Toward Improvement of Charge Injection and Ionic Conduction in Organic Mixed Ionic–Electronic Conductors for Organic Electrochemical Transistors,” Chemical Reviews 122, no. 4 (2022): 4325–4355, 10.1021/acs.chemrev.1c00266. PMC8874907 34902244

[4] A. Nawaz , Q. Liu , W. L. Leong , K. E. Fairfull‐Smith , and P. Sonar , “Organic Electrochemical Transistors for In Vivo Bioelectronics,” Advanced Materials 33 (2021): 202101874, 10.1002/adma.202101874. 34606146

[5] Y. Yao , R. M. Pankow , W. Huang , et al., “An Organic Electrochemical Neuron for a Neuromorphic Perception System,” Proceedings of the National Academy of Sciences 122, no. 2 (2025): 2414879122, 10.1073/pnas.2414879122. PMC11745397 39773026

[6] J. Rivnay , S. Inal , A. Salleo , R. M. Owens , M. Berggren , and G. G. Malliaras , “Organic Electrochemical Transistors,” Nature Reviews Materials 3, no. 2 (2018): 17086, 10.1038/natrevmats.2017.86.

[7] Y. Yao , W. Huang , J. Chen , et al., “Flexible Complementary Circuits Operating at Sub‐0.5 V via Hybrid Organic–Inorganic Electrolyte‐Gated Transistors,” Proceedings of the National Academy of Sciences 118, no. 44 (2021): 2111790118, 10.1073/pnas.2111790118. PMC8612221 34716274

[8] Y. Yao , W. Huang , J. Chen , et al., “Flexible and Stretchable Organic Electrochemical Transistors for Physiological Sensing Devices,” Advanced Materials 35, no. 35 (2023): 202209906, 10.1002/adma.202209906. 36808773

[9] P. Oldroyd , S. Velasco‐Bosom , S. L. Bidinger , T. Hasan , A. J. Boys , and G. G. Malliaras , “Fabrication of Thin‐Film Electrodes and Organic Electrochemical Transistors for Neural Implants,” Nature Protocols 20, no. 8 (2025): 2100–2124, 10.1038/s41596-024-01116-6. 39934461

[10] Y. Liu , X. Su , P. Fan , X. Liu , Y. Pan , and J. Ping , “Computationally‐Assisted Wearable System for Continuous Cortisol Monitoring,” Science Bulletin 70, no. 12 (2025): 2004–2013, 10.1016/j.scib.2025.03.060. 40328603

[11] Y. Zheng , S. Zhang , J. B.‐H. Tok , and Z. Bao , “Molecular Design of Stretchable Polymer Semiconductors: Current Progress and Future Directions,” Journal of the American Chemical Society 144, no. 11 (2022): 4699–4715, 10.1021/jacs.2c00072. 35262336

[12] J. Y. Oh , S. Rondeau‐Gagné , Y.‐C. Chiu , et al., “Intrinsically Stretchable and Healable Semiconducting Polymer for Organic Transistors,” Nature 539, no. 7629 (2016): 411–415, 10.1038/nature20102. 27853213

[13] J. Xu , S. Wang , G.‐J. N. Wang , et al., “Highly Stretchable Polymer Semiconductor Films Through the Nanoconfinement Effect,” Science 355, no. 6320 (2017): 59–64, 10.1126/science.aah4496. 28059762

[14] J. Chen , W. Huang , D. Zheng , et al., “Highly Stretchable Organic Electrochemical Transistors With Strain‐Resistant Performance,” Nature Materials 21, no. 5 (2022): 564–571, 10.1038/s41563-022-01239-9. 35501364

[15] H. F. de , P. Barbosa , A. Asyuda , M. Skowrons , A. Schander , and B. Lüssem , “Processing of Organic Electrochemical Transistors,” MRS Communications 14 (2024): 132–148, 10.1557/s43579-024-00521-y.

[16] S. Chen , A. Surendran , X. Wu , S. Y. Lee , M. Stephen , and W. L. Leong , “Recent Technological Advances in Fabrication and Application of Organic Electrochemical Transistors,” Advanced Materials Technologies 5, no. 12 (2020): 202000523, 10.1002/admt.202000523.

[17] Z. Wang , K. Chen , X. Shi , et al., “Fibre Integrated Circuits by a Multilayered Spiral Architecture,” Nature 650, no. 8100 (2026): 102–109, 10.1038/s41586-025-09974-0. 41565807

[18] M. Zhu , J. Li , J. Yu , Z. Li , and B. Ding , “Superstable and Intrinsically Self‐Healing Fibrous Membrane With Bionic Confined Protective Structure for Breathable Electronic Skin,” Angewandte Chemie 134, no. 22 (2022): 202200226, 10.1002/ange.202200226. 35212123

[19] Z. Chang , F. Wang , Z. Wang , J. Yu , B. Ding , and Z. Li , “Fiber‐Based Electrochemical Sweat Sensors Toward Personalized Monitoring,” Progress in Materials Science 156 (2026): 101579, 10.1016/j.pmatsci.2025.101579.

[20] F. Sun , H. Jiang , H. Wang , et al., “Soft Fiber Electronics Based on Semiconducting Polymer,” Chemical Reviews 123, no. 8 (2023): 4693–4763, 10.1021/acs.chemrev.2c00720. 36753731

[21] B. Fang , J. Yan , D. Chang , et al., “Scalable Production of Ultrafine Polyaniline Fibres for Tactile Organic Electrochemical Transistors,” Nature Communications 13, no. 1 (2022): 2101, 10.1038/s41467-022-29773-9. PMC9018749 35440125

[22] P. Alarcon‐Espejo , R. Sarabia‐Riquelme , G. M. Matrone , et al., “High‐Hole‐Mobility Fiber Organic Electrochemical Transistors for Next‐Generation Adaptive Neuromorphic Bio‐Hybrid Technologies,” Advanced Materials 36, no. 11 (2024): 202305371, 10.1002/adma.202305371. 37824715

[23] W. Chen , H. Jiang , C. Du , et al., “Fiber Transistors as a Hardware Surrogate Gradient for Backpropagation in Spiking Neural Networks,” Advanced Materials 38, no. 6 (2026): 202514904, 10.1002/adma.202514904. 41195912

[24] X. Wang , H. Jiang , H. Wang , et al., “Shear‐Intensified Hybridization of Conjugated Polymer Fibers for Organic Electrochemical Transistors,” Advanced Functional Materials 36, no. 14 (2026): 202515197, 10.1002/adfm.202515197.

[25] Y. Dai , S. Dai , N. Li , et al., “Stretchable Redox‐Active Semiconducting Polymers for High‐Performance Organic Electrochemical Transistors,” Advanced Materials 34, no. 23 (2022): 202201178, 10.1002/adma.202201178. 35448913

[26] N. Li , Y. Li , Z. Cheng , et al., “Bioadhesive Polymer Semiconductors and Transistors for Intimate Biointerfaces,” Science 381, no. 6658 (2023): 686–693, 10.1126/science.adg8758. PMC10768720 37561870

[27] C. B. Nielsen , A. Giovannitti , D.‐T. Sbircea , et al., “Molecular Design of Semiconducting Polymers for High‐Performance Organic Electrochemical Transistors,” Journal of the American Chemical Society 138, no. 32 (2016): 10252–10259, 10.1021/jacs.6b05280. PMC4991841 27444189

[28] J. Xu , H.‐C. Wu , C. Zhu , et al., “Multi‐Scale Ordering in Highly Stretchable Polymer Semiconducting Films,” Nature Materials 18, no. 6 (2019): 594–601, 10.1038/s41563-019-0340-5. 30988452

[29] Z. Zhang , P. Li , M. Xiong , et al., “Continuous Production of Ultratough Semiconducting Polymer Fibers With High Electronic Performance,” Science Advances 10, no. 14 (2024): adk0647, 10.1126/sciadv.adk0647. PMC10990280 38569023

[30] K. Liu , J. Wang , X. Pan , et al., “N‐Type Thermoelectric Elastomers,” Nature 644, no. 8078 (2025): 920–926, 10.1038/s41586-025-09387-z. 40804523

[31] B. O'Connor , E. P. Chan , C. Chan , et al., “Correlations Between Mechanical and Electrical Properties of Polythiophenes,” ACS Nano 4, no. 12 (2010): 7538–7544, 10.1021/nn1018768. 21080648

[32] R. Peng , B. Pang , D. Hu , et al., “An ABA Triblock Copolymer Strategy for Intrinsically Stretchable Semiconductors,” Journal of Materials Chemistry C 3, no. 15 (2015): 3599–3606, 10.1039/C4TC02476A.

[33] J. Mun , G.‐J. N. Wang , J. Y. Oh , et al., “Effect of Nonconjugated Spacers on Mechanical Properties of Semiconducting Polymers for Stretchable Transistors,” Advanced Functional Materials 28, no. 43 (2018): 201804222, 10.1002/adfm.201804222.

[34] G.‐J. N. Wang , Y. Zheng , S. Zhang , et al., “Tuning the Cross‐Linker Crystallinity of a Stretchable Polymer Semiconductor,” Chemistry of Materials 31, no. 17 (2019): 6465–6475, 10.1021/acs.chemmater.8b04314.

[35] Y.‐C. Chiang , H.‐C. Wu , H.‐F. Wen , et al., “Tailoring Carbosilane Side Chains Toward Intrinsically Stretchable Semiconducting Polymers,” Macromolecules 52, no. 11 (2019): 4396–4404, 10.1021/acs.macromol.9b00589.

[36] J. Mun , J. Kang , Y. Zheng , et al., “Conjugated Carbon Cyclic Nanorings as Additives for Intrinsically Stretchable Semiconducting Polymers,” Advanced Materials 31, no. 42 (2019): 201903912, 10.1002/adma.201903912. 31489716

[37] G.‐J. N. Wang , L. Shaw , J. Xu , et al., “Inducing Elasticity Through Oligo‐Siloxane Crosslinks for Intrinsically Stretchable Semiconducting Polymers,” Advanced Functional Materials 26, no. 40 (2016): 7254–7262, 10.1002/adfm.201602603.

[38] J. Mun , Y. Ochiai , W. Wang , et al., “A Design Strategy for High Mobility Stretchable Polymer Semiconductors,” Nature Communications 12, no. 1 (2021): 3572, 10.1038/s41467-021-23798-2. PMC8196107 34117254

[39] D. Liu , X. Tian , J. Bai , et al., “Intrinsically Stretchable Organic Electrochemical Transistors With Rigid‐Device‐Benchmarkable Performance,” Advanced Science 9, no. 29 (2022): 202203418, 10.1002/advs.202203418. PMC9561867 35904088

[40] X. Yu , L. Chen , C. Li , et al., “Intrinsically Stretchable Polymer Semiconductors With Good Ductility and High Charge Mobility Through Reducing the Central Symmetry of the Conjugated Backbone Units,” Advanced Materials 35, no. 17 (2023): 202209896, 10.1002/adma.202209896. 36772843

[41] M. Shin , J. Y. Oh , K.‐E. Byun , et al., “Polythiophene Nanofibril Bundles Surface‐Embedded in Elastomer: A Route to a Highly Stretchable Active Channel Layer,” Advanced Materials 27, no. 7 (2015): 1255–1261, 10.1002/adma.201404602. 25581228

[42] S. Zhang , Y. Cheng , L. Galuska , et al., “Tacky Elastomers to Enable Tear‐Resistant and Autonomous Self‐Healing Semiconductor Composites,” Advanced Functional Materials 30, no. 27 (2020): 202000663, 10.1002/adfm.202000663.

[43] J. Zuo , T. Jin , H. Li , et al., “Continuous Conjugated Polymer Network in Elastomer Matrix Arising From Solution‐State Aggregation and Film‐Forming Dynamics Favoring Mechanical and Electrical Properties,” Advanced Functional Materials 35, no. 28 (2025): 202424785, 10.1002/adfm.202424785.

[44] C. Gao , K. Chenchai , L. Huang , et al., “Polymer Semiconductor Blends With Remarkably Stable Semiconducting Performance Under Large and Cyclic Mechanical Deformation,” Nature Communications 16, no. 1 (2025): 10388, 10.1038/s41467-025-65327-5. PMC12644822 41285719

[45] X. Wang , Z. Zhang , P. Li , et al., “Ultrastable N‐Type Semiconducting Fiber Organic Electrochemical Transistors for Highly Sensitive Biosensors,” Advanced Materials 36, no. 24 (2024): 202400287, 10.1002/adma.202400287. 38433667

[46] H. Jiang , C.‐Y. Yang , D. Tu , et al., “Shear‐Enhanced Liquid‐Crystal Spinning of Conjugated Polymer Fibers,” National Science Review 12, no. 10 (2025): nwaf331, 10.1093/nsr/nwaf331. PMC12485984 41040493

[47] S. Ma , J. Wang , W. Wu , et al., “Sequential Cyanation of Polythiophenes: Tuning Charge Carrier Polarity in Organic Electrochemical Transistors,” Advanced Electronic Materials 9, no. 11 (2023): 202300207, 10.1002/aelm.202300207.

[48] W. Yang , S. Ma , S. Gámez‐Valenzuela , et al., “Backbone Tailoring Enables High‐Performance and Stable N‐Type Organic Mixed Ionic‐Electronic Conductors for Synaptic Simulation and Biosensor,” Advanced Materials 38, no. 3 (2026): 202512070, 10.1002/adma.202512070. 41045119

[49] Z. Wang , Y. Wu , Q. Zhou , et al., “Aggregation Structure and Glass Transition of Intrinsically Stretchable Semiconducting Polymers,” Matter 6, no. 10 (2023): 3434–3448, 10.1016/j.matt.2023.05.042.

[50] J. Li , J. Cai , J. Yu , Z. Li , and B. Ding , “The Rising of Fiber Constructed Piezo/Triboelectric Nanogenerators: From Material Selections, Fabrication Techniques to Emerging Applications,” Advanced Functional Materials 33, no. 44 (2023): 202303249, 10.1002/adfm.202303249.

[51] Z. Xu , C. Zhang , F. Wang , et al., “Smart Textiles for Personalized Sports and Healthcare,” Nano‐Micro Letters 17, no. 1 (2025): 232, 10.1007/s40820-025-01749-6. PMC12031719 40278986

[52] Y. Wu , F. Wang , Z. Wang , et al., “Highly Stretchable, Resistance‐Stable, Conductive Liquid Metal Core‐Sheath Fibers Enable Ultrastable and Supersensitive Physiological Monitoring,” Small 21, no. 42 (2025): 202507439, 10.1002/smll.202507439. 40898662

[53] Q. Zhou , Z. Wang , Y. Yan , et al., “Strain‐Enhanced Electrical Performance in Stretchable Semiconducting Polymers,” NPJ Flexible Electronics 7, no. 1 (2023): 35, 10.1038/s41528-023-00269-w.

[54] J. Rivnay , S. C. B. Mannsfeld , C. E. Miller , A. Salleo , and M. F. Toney , “Quantitative Determination of Organic Semiconductor Microstructure From the Molecular to Device Scale,” Chemical Reviews 112, no. 10 (2012): 5488–5519, 10.1021/cr3001109. 22877516

[55] A. M. Hindeleh and R. Hosemann , “Paracrystals Representing the Physical State of Matter,” Journal of Physics C: Solid State Physics 21, no. 23 (1988): 4155–4170, 10.1088/0022-3719/21/23/004.

[56] M. Ponçot , J. Martin , S. Chaudemanche , et al., “Complementarities of High Energy WAXS and Raman Spectroscopy Measurements to Study the Crystalline Phase Orientation in Polypropylene Blends During Tensile Test,” Polymer 80 (2015): 27–37, 10.1016/j.polymer.2015.10.040.

[57] Z. Zhu , X. Wang , D. Wang , et al., “Fine‐Grained ECG Monitoring Based on Stretchable Organic Electrochemical Transistors With Anisotropic Gel Electrolytes for Biometric Identification,” ACS Sensors 10, no. 12 (2025): 9423–9431, 10.1021/acssensors.5c02403. 41324981

[58] Y. Pang , Y. Li , Y. Gu , et al., “Stretchable Organic Electrochemical Transistors for Sustained High‐Fidelity Electrophysiology and Deep Learning‐Assisted Sleep Monitoring,” Matter 8, no. 5 (2025): 102086, 10.1016/j.matt.2025.102086.

