# Supplementary material for: Strain‐Enhanced Ultrastretchable Semiconducting Polymer Fibers for Organic Electrochemical Transistors
Source: Adv Sci (Weinh). 2026 Nov 5:e78666. Online ahead of print. doi: 10.1002/advs.78666 (PMC13735436; doi:10.1002/advs.78666)
Supplement: Supplementary file 1 [file ADVS-9999-e78666-s002.docx]

**Supplementary Information for**

**Strain**-**enhanced Ultrastretchable Semiconducting Polymer Fibers for Organic Electrochemical Transistors**

Zixu Chang^1^, Ziao Xu^1^, Qi Tang^1^, Mingjuan Du^1^, Faqiang Wang^1^, Jiexun Zhu^1^, Jianyong Yu^2^, Bin Ding^2^, Zhaoling Li^1,3^

^1^Shanghai Frontiers Science Center of Advanced Textiles, College of Textiles, Donghua University, Shanghai, China

^2^Innovation Center for Textile Science and Technology, Donghua University, Shanghai, China

^3^Jiangsu Laboratory of Advanced Functional Materials, School of Materials Engineering, Suzhou University of Technology, Suzhou 215500, China.

**Correspondence**: Zhaoling Li (zli@dhu.edu.cn) | Bin Ding (binding@dhu.edu.cn)

**Figure S1.** The p(g2T-T) solution can be wet spun into fibers at different concentrations, coagulation baths, and needle sizes. a-i are optical photos of fibers under different spinning parameters. The image on the left shows an optical image of the fibers in the coagulation bath, while the image on the right shows the corresponding fibers after they were removed from the coagulation bath and dried. D is the diameter of the needle, and L is the length of the needle. Compared to other coagulation baths, p(g2T-T) fibers coagulated in ethanol exhibit the best uniformity.

**Optimization of Wet Spinning Process**

In order to obtain ideal p(g2T-T) fibers, we optimized the spinning process parameters, including concentration (**Figure S2**), shear speed (**Figure S3**), spinning needle size, and coagulation bath (**Figure S4)**.

**Concentration:** Solutions with higher concentrations typically exhibit higher viscosity, which increases the shear stress required to align the conjugated polymer chains. Furthermore, high concentrations introduce aggregates into the solution, which facilitate better orientation through solution shearing. Since polymers exhibit an entangled state only at concentrations above the critical overlap concentration (reported in the literature to be around 5 mg mL^-1^) ^[1]^, the lowest concentration selected for our experiments was 5 mg mL^-1^. However, semiconducting conjugated polymers typically have limited solubility and tend to aggregate strongly in a disordered manner in solution. Excessively high concentrations not only make the polymer difficult to dissolve but also lead to excessive aggregation of the solution during the spinning process, preventing fiber formation. We found that the polymer could still dissolve completely at around 15 mg mL^-1^. Therefore, we compared fibers at different concentrations ranging from 5 mg mL^-1^ to 15 mg mL^-1^. The morphology of p(g2T-T) fibers is characterized using SEM and UV-vis spectroscopy. As the concentration increases, the alignment gradually improves, and the mechanical properties and electrical conductivity of the fibers also improve (electrical conductivity is roughly estimated using the surface resistivity of the fibers). The error bars for mechanical properties represent the standard deviation obtained from 3 measurements of at least 2 different samples under each condition. The error bars for electrical conductivity represent the standard deviation obtained from 6 measurements.

**Figure S2.** The effect of solution concentration on p(g2T-T) fibers. (a) SEM images of p(g2T-T) fibers fabricated from solutions of different concentrations. (b) The UV-vis absorption spectra of solutions of p(g2T-T) at different concentrations. (c) The mechanical properties of p(g2T-T) fibers at different concentrations. (d) The conductivity of p(g2T-T) fibers at different concentrations.

**Shear speed:** Fibers with low shear rates tend to have high surface roughness, which is detrimental to good mechanical stretching. Therefore, we conducted spinning at shear speeds ranging from 0.3, 0.6, 0.9, to 1.2 ml/h. To improve fiber formation from the solution, we aimed to allow the entangled polymer to depolymerize under shear flow. At a certain speed (the critical injection speed), the absorbance of the polymer solution drops sharply (**Figure S3b**), indicating significant depolymerization. Although further increasing the shear speed can generate greater shear stress in the solution, higher spinning speeds result in coarser fibers in the coagulation bath. When the fibers are subsequently removed from the solidification bath and dried, this may lead to residual solvent, which further affects surface smoothness and causes the fiber structure to become loose. Consequently, a spinning speed of 0.9 ml/h is appropriate.

**Figure S3.** The effect of shear speed on p(g2T-T) fibers. (a) SEM images of p(g2T-T) fibers fabricated from different shear speed. (b) The UV-vis absorption spectra of solutions of p(g2T-T) at different shear speed. (c) The mechanical properties of p(g2T-T) fibers at different shear speed. (d) The conductivity of p(g2T-T) fibers at different shear speed.

**Needle size:** Using a small-diameter, long needle generates a high strain rate, which unravels entangled polymer chains and aligns them in the direction of flow. Reducing the needle diameter or increasing the needle length enhances the alignment of polymer chains within the fiber. In preliminary experiments, we found that fibers spun using needles with large inner diameters were non-uniform. Therefore, unless otherwise specified, the needle used in this experiment has a diameter of 60 μm and a length of 38 mm.

**Coagulation bath:** We use a coagulation bath to induce phase separation and crystallization in the depolymerized polymer. The polarity of the solidification bath is crucial for regulating the crystallinity and chain orientation of the semiconductor polymer fibers. This phase separation process is related to diffusion within the solidification bath and can be explained using Hansen solubility parameters. In our previous investigations, we found that this polymer can be fiberized in up to eight different coagulation baths. To select an appropriate coagulation bath, we first conducted a preliminary screening using Hansen solubility theory (isopropanol, ethanol, acetonitrile), then compared the various properties of the fibers, and ultimately decided to use ethanol as the coagulation bath after comprehensive consideration.

**Figure S4.** The effect of coagulation bath on p(g2T-T) fibers. (a) SEM images of p(g2T-T) fibers fabricated from different coagulation bath. (b) The mechanical properties of p(g2T-T) fibers at different coagulation bath. (c) The conductivity of p(g2T-T) fibers at different coagulation bath.

**Figure S5.** The effect of different needle sizes and injection speeds on aggregate size in polymer solutions. (a) Particle size of aggregates at different needle sizes and shear rates. (b) Dynamic light scattering (DLS) analysis of p(g2T-T) solutions extruded from different needles. (c) DLS of p(g2T-T) solutions at different injection speeds.

**Figure S6.** Comparison of solubility parameters for chloroform and several coagulation baths. (a) Contributions of each component to the solubility parameters of chloroform and the coagulation bath. (b) Comparison of the total solubility parameter δ between chloroform and the coagulation bath to show the energy difference Δδ.

**Figure S7.** Polarized optical microscopy images of the stretched p(g2T-T) fiber.

**Figure S8.** Photoluminescence spectra of the p(g2T-T) solution, film and fiber.

**Figure S9.** CV measurements of fibers and films. (a) CV curves of p(g2T-T) films and fibers in NaCl and acetonitrile solutions, respectively. (b) CV curves of p(g2T-T) films and fibers in NaCl. (c) CV curves of ferrocene in acetonitrile electrolyte for calibration.

**Figure S10.** CV scans of (a) p(g2T-T) films, and (b) p(g2T-T) fibers in NaCl solutions and (c) p(g2T-T) films, and (d) p(g2T-T) fibers in acetonitrile solutions over 30 cycles.

**Figure S11.** Operando UV-vis measurements on a p(g2T-T) film.

**Figure S12.** Comparison of transfer characteristics of fibers and films. (a) Transfer characteristics and corresponding curves of p(g2T-T) fiber OECT. (b) Transfer characteristics and corresponding *g*_m_ curves of p(g2T-T) film OECT.

**Figure S13.** Electrochemical impedance spectroscopy measurement results for (a) the Nyquist plot of a p(g2T-T) film, (b) the Bode plot of a p(g2T-T) film, (c) the Nyquist plot of a p(g2T-T) fiber, and (d) the Bode plot of a p(g2T-T) fiber.

**Figure S14.** Stress-strain curves of p(g2T-T) fiber at 20% stretch-recovery cycle test.

**Figure S15.** Stretching performance of p(g2T-T) fibers. (a) Photographs of p(g2T-T) fibers stretching from 0% to 250%. (b) Photographs showing p(g2T-T) fibers being stretched from 0% to 250% on a tensile testing machine, as well as photographs of the fibers embedded in PDMS during stretching. After being stretched to 250%, the fibers did not break and exhibited a buckled shape after recovery.

**Figure S16.** Output curves of p(g2T-T) fiber OECTs under (a) 0%, (b) 50%, (c) 100%, (d) 150%, (e) 200%, and (f) 250% strains.

**Figure S17.** Transfer curves and (-*I*_d_)^1/2^ of p(g2T-T) fiber OECTs under different strains.

**Figure S18.** CV curves of stretched p(g2T-T) fibers under different strains.

**Figure S19.** The (a) Nyquist and (b) Bode plots of p(g2T-T) fibers under different strains.

**Figure S20.** Transient responses of p (g2T-T) fiber OECTs under different strains.

**Figure S21.** Stability evaluation of p(g2T-T) fiber OECTs. (a) Transfer curves and *g*_m_ of p(g2T-T) fiber OECTs under 1,000 stretching cycles to 200% strain. (b) Changes of the *g*_m, normalized_, *μC**, and *µ* of the p(g2T-T) fiber OECTs during 1,000 stretching cycles to 200% strain.

**Figure S22.** The lamellar distance of the p(g2T-T) fiber under different strains.

**Figure S23.** The azimuthal intensity plot of the (100) reflection of p(g2T-T) fibers under different strains.

**Figure S24.** Sheet resistance of stretchable silver paste under (a) strain and (b) stretching cycle.

**Figure S25.** Transfer curve and corresponding *g*_m_ curve of the fully stretchable fiber OECT.

**Figure S26.** Transfer curves and *g*_m_ curves of stretchable fiber OECTs under different strains.

**Figure S27.** Output curves of stretchable fiber OECTs under (a) 0%, (b) 50%, (c) 100%, (d) 150%, (e) 200%, and (f) 250% strains.

**Figure S28.** Transfer curves and corresponding *g*_m_ curves of the stretchable fiber OECT under (a) stretched and (b) released state during the stretching cycle measurement.

**Figure S29.** ECG signals recorded by the fully stretchable fiber OECT under different strains.

**Table S1.** p(g2T-T) conjugated polymers characteristics

| **Batch of polymer** | **Mn (kDa)** | **Mw (kDa)** | **PDI** |
| --- | --- | --- | --- |
| 1 | 76.6 | 118.6 | 1.55 |
| 2 | 62.0 | 107.1 | 1.73 |
| 3 | 70.4 | 112.2 | 1.59 |

**Table S2.** Hansen solubility parameters^*^ for various solvents

| **Number** | **Solvent** | ***δ*** | ***δ*_D_** | ***δ*_P_** | ***δ*_H_** |
| --- | --- | --- | --- | --- | --- |
| 1 | CHCl_3_ | 19.0 | 17.8 | 3.1 | 5.7 |
| 2 | TL | 18.2 | 18.0 | 1.4 | 2.0 |
| 3 | *n*BA | 17.4 | 15.8 | 3.7 | 6.3 |
| 4 | THF | 19.4 | 16.8 | 5.7 | 8 |
| 5 | IPA | 23.6 | 15.8 | 6.1 | 16.4 |
| 6 | EtOH | 26.5 | 15.8 | 8.8 | 19.4 |
| 7 | ACE | 19.9 | 15.5 | 10.4 | 7 |
| 8 | MeOH | 29.6 | 15.1 | 12.3 | 22.3 |
| 9 | ACN | 24.4 | 15.3 | 18.0 | 6.1 |

* The Hansen solubility parameter consists of three main components: the dispersion contribution (*δ*_D_), the polarity contribution (*δ*_P_), and the hydrogen-bonding contribution (*δ*_H_). The total solubility parameter (*δ*) is calculated using the following equation: $\delta=\sqrt{{\delta_{D}}^{2}+{\delta_{P}}^{2}+{\delta_{H}}^{2}}$

**Table S3.** Summary of the charge carrier mobility, modulus, and strain of the representative stretchable semiconductors.

| **Semiconductor type** | **Material** | **Mobility (cm^2^ V^-1^ s^-1^)** | **Young’s modulus** | **Strain** | **Reference** |
| --- | --- | --- | --- | --- | --- |
| Molecular structure design of soft and stretchable polymer semiconductor films | P3HT | 0.06 | 250 MPa | 150% | ^[2]^ |
|  | P3HT-PMA-P3HT | 9×10^-4^ | 6 MPa | 140% | ^[3]^ |
|  | P(DPP-TT) | 0.66 | ~ 100 MPa | 150% | ^[4]^ |
|  | P(DPPTVT)-PDCA | 1.3 | ~ 400 MPa | ~ 100% | ^[5]^ |
|  | DPP-TVT-C12 | ~ 1 | 130 MPa | 100% | ^[6]^ |
|  | DPP-10C_5_DE | 0.2 | ~ 200 MPa | 100% | ^[7]^ |
|  | P(DPPTT)-CPP | 0.75 | 110 MPa | 105% | ^[8]^ |
|  | PII2T-C10 | ~ 1 | 250 MPa | 100% | ^[9]^ |
|  | P(DPPTVT) | ~ 1 | ~ 550 MPa | ~ 100% | ^[10]^ |
|  | PEDOT: PSS | ~ 1.1 | ~ 20 MPa | ~ 50% | ^[11]^ |
|  | P(g2T-T) | ~ 1.7 | 50 MPa | 200% | ^[12]^ |
|  | P(DPP-g2T) | ~ 1.69 | ~ 30 MPa | 140% | ^[13]^ |
|  | P(DPP-BT-piro[cycloalkane-1,9′fluorene]) | 3.1 | 83.7 MPa | 150% | ^[14]^ |
| Polymer semiconductor films blended with elastomers | P3HT/SEBS | 0.007 | 11.7 MPa | 50% | ^[15]^ |
|  | P(DPPTT)/SEBS | ~ 1 | ~ 20 MPa | 100% | ^[16]^ |
|  | PDPPTVT/Butyl rubber | 1 | 1 MPa | 150% | ^[17]^ |
|  | PDPPT3/SEBS | 0.99 | ~ 10 MPa | 177% | ^[18]^ |
|  | PDPPTT/H-PIP | 1.8 | 6.5 MPa | 100% | ^[19]^ |
| Polymer semiconductor fibers | BBL | ~ 0.021 | ~ 6.1 GPa | 6.8% | ^[20]^ |
|  | BBL | ~ 0.011 | ~ 6.9 GPa | ~ 15% | ^[21]^ |
|  | BBL/GO | ~ 0.018 | ~ 6.7 GPa | ~ 2% | ^[22]^ |
|  | P(g2T-T) | 1.41 | 32.4 MPa | > 250% | This work |

**Table S4.** Optical and electrochemical properties of p(g2T-T) films and fibers

| **P(g2T-T)** | ***λ*^sol^_max_**  (nm) | ***λ_m_*_ax_**  (nm) | ***λ*_onset_**  (nm) | ***E*^opt^_g_**  (eV) | ***E*_LUMO_**  (eV) | ***E*_HOMO_^*^**  (eV) |
| --- | --- | --- | --- | --- | --- | --- |
| film | 575 | 609 | 726 | 1.708 | -2.62 | -4.33 |
| fiber | 575 | 663 | 741 | 1.673 | -2.89 | -4.56 |

**E*_HOMO_ is calculated using the equation - *E*_HOMO_ = (*E*_OX_ - *E*_FC/FC+_ + 4.8) eV. *E*_OX_ is the onset of oxidation peak, extracted from Figure S9b, and *E*_FC/FC+_ is extracted from Figure S9c. *E*_LUMO_ is calculated using the equation *E*_LUMO_ = *E*_HOMO_ + *E*^opt^_g_. *E*^opt^_g_ is calculated using the equation *E*^opt^_g_ = 1240/*λ*_onset_, where *λ*_onset_ was extracted from the UV absorption spectra ^[23,24]^.

**Table S5.** Comparison of molecular packing parameters (*g* factor, *Lc*, *d*-spacing, and Herman’s orientation factor (*f*)) of p(g2T-T) fibers under different strains.

| **P(g2T-T) fiber** | **(100) peak** | | | | **(010) peak** | | | | ***d*_lamellar_ (100)** | ***d*_π-π_ (010)** | **FWHM** | **Herman’s orientation**  **factor (*f*)** |
| --- | --- | --- | --- | --- | --- | --- | --- | --- | --- | --- | --- | --- |
|  | ***Δq*** | ***q_0_*** | ***g* factor** | ***Lc*** | ***Δq*** | ***q*_0_** | ***g* factor** | ***Lc*** |  |  | **(*ψ*)** |  |
| 0% stretched | 0.155 | 0.377 | 0.255 | 36.078 | 0.201 | 1.708 | 0.137 | 27.821 | 16.666 | 3.679 | 60.346 | 0.665 |
| 50% stretched | 0.154 | 0.383 | 0.253 | 36.312 | 0.196 | 1.735 | 0.134 | 28.530 | 16.405 | 3.621 | 42.098 | 0.766 |
| 100% stretched | 0.146 | 0.406 | 0.239 | 38.302 | 0.190 | 1.737 | 0.132 | 29.432 | 15.476 | 3.617 | 34.760 | 0.807 |
| 150% stretched | 0.145 | 0.413 | 0.236 | 38.566 | 0.173 | 1.741 | 0.126 | 32.324 | 15.214 | 3.609 | 31.006 | 0.828 |
| 200% stretched | 0.138 | 0.419 | 0.229 | 40.522 | 0.161 | 1.743 | 0.121 | 34.733 | 14.996 | 3.605 | 25.960 | 0.856 |
| 250% stretched | 0.137 | 0.423 | 0.227 | 40.818 | 0.159 | 1.749 | 0.120 | 35.170 | 14.854 | 3.592 | 24.731 | 0.863 |

**Movie S1.** The deformation behavior of the p(g2T-T) fiber under tensile stress.

**References**

1. R. Venkatesh, Y. Zheng, C. Viersen, et al., “Data Science Guided Experiments Identify Conjugated Polymer Solution Concentration as a Key Parameter in Device Performance,” *ACS Materials Letters* *3*, no. 9 (2021): 1321–1327, https://doi.org/10.1021/acsmaterialslett.1c00320.

2. B. O’Connor, E. P. Chan, C. Chan, et al., “Correlations between Mechanical and Electrical Properties of Polythiophenes,” *ACS Nano* *4*, no. 12 (2010): 7538–7544, https://doi.org/10.1021/nn1018768.

3. R. Peng, B. Pang, D. Hu, et al., “An ABA Triblock Copolymer Strategy for Intrinsically Stretchable Semiconductors,” *Journal of Materials Chemistry C* *3*, no. 15 (2015): 3599–3606, https://doi.org/10.1039/C4TC02476A.

4. G. N. Wang, L. Shaw, J. Xu, et al., “Inducing Elasticity through Oligo‐Siloxane Crosslinks for Intrinsically Stretchable Semiconducting Polymers,” *Advanced Functional Materials* *26*, no. 40 (2016): 7254–7262, https://doi.org/10.1002/adfm.201602603.

5. J. Y. Oh, S. Rondeau-Gagné, Y.-C. Chiu, et al., “Intrinsically Stretchable and Healable Semiconducting Polymer for Organic Transistors,” *Nature* *539*, no. 7629 (2016): 411–415, https://doi.org/10.1038/nature20102.

6. J. Mun, G. N. Wang, J. Y. Oh, et al., “Effect of Nonconjugated Spacers on Mechanical Properties of Semiconducting Polymers for Stretchable Transistors,” *Advanced Functional Materials* *28*, no. 43 (2018), https://doi.org/10.1002/adfm.201804222.

7. G.-J. N. Wang, Y. Zheng, S. Zhang, et al., “Tuning the Cross-Linker Crystallinity of a Stretchable Polymer Semiconductor,” *Chemistry of Materials* *31*, no. 17 (2019): 6465–6475, https://doi.org/10.1021/acs.chemmater.8b04314.

8. J. Mun, J. Kang, Y. Zheng, et al., “Conjugated Carbon Cyclic Nanorings as Additives for Intrinsically Stretchable Semiconducting Polymers,” *Advanced Materials* *31*, no. 42 (2019), https://doi.org/10.1002/adma.201903912.

9. Y.-C. Chiang, H.-C. Wu, H.-F. Wen, et al., “Tailoring Carbosilane Side Chains toward Intrinsically Stretchable Semiconducting Polymers,” *Macromolecules* *52*, no. 11 (2019): 4396–4404, https://doi.org/10.1021/acs.macromol.9b00589.

10. J. Mun, Y. Ochiai, W. Wang, et al., “A Design Strategy for High Mobility Stretchable Polymer Semiconductors,” *Nature Communications* *12*, no. 1 (2021): 3572, https://doi.org/10.1038/s41467-021-23798-2.

11. D. Liu, X. Tian, J. Bai, et al., “Intrinsically Stretchable Organic Electrochemical Transistors with Rigid‐Device‐Benchmarkable Performance,” *Advanced Science* *9*, no. 29 (2022), https://doi.org/10.1002/advs.202203418.

12. Y. Dai, S. Dai, N. Li, et al., “Stretchable Redox‐Active Semiconducting Polymers for High‐Performance Organic Electrochemical Transistors,” *Advanced Materials* *34*, no. 23 (2022), https://doi.org/10.1002/adma.202201178.

13. J. Chen, W. Huang, D. Zheng, et al., “Highly Stretchable Organic Electrochemical Transistors with Strain-Resistant Performance,” *Nature Materials* *21*, no. 5 (2022): 564–571, https://doi.org/10.1038/s41563-022-01239-9.

14. X. Yu, L. Chen, C. Li, et al., “Intrinsically Stretchable Polymer Semiconductors with Good Ductility and High Charge Mobility through Reducing the Central Symmetry of the Conjugated Backbone Units,” *Advanced Materials* *35*, no. 17 (2023), https://doi.org/10.1002/adma.202209896.

15. M. Shin, J. Y. Oh, K. Byun, et al., “Polythiophene Nanofibril Bundles Surface‐Embedded in Elastomer: A Route to a Highly Stretchable Active Channel Layer,” *Advanced Materials* *27*, no. 7 (2015): 1255–1261, https://doi.org/10.1002/adma.201404602.

16. J. Xu, S. Wang, G.-J. N. Wang, et al., “Highly Stretchable Polymer Semiconductor Films through the Nanoconfinement Effect,” *Science* *355*, no. 6320 (2017): 59–64, https://doi.org/10.1126/science.aah4496.

17. S. Zhang, Y. Cheng, L. Galuska, et al., “Tacky Elastomers to Enable Tear‐Resistant and Autonomous Self‐Healing Semiconductor Composites,” *Advanced Functional Materials* *30*, no. 27 (2020), https://doi.org/10.1002/adfm.202000663.

18. J. Zuo, T. Jin, H. Li, et al., “Continuous Conjugated Polymer Network in Elastomer Matrix Arising from Solution‐State Aggregation and Film‐Forming Dynamics Favoring Mechanical and Electrical Properties,” *Advanced Functional Materials* *35*, no. 28 (2025), https://doi.org/10.1002/adfm.202424785.

19. C. Gao, K. Chenchai, L. Huang, et al., “Polymer Semiconductor Blends with Remarkably Stable Semiconducting Performance under Large and Cyclic Mechanical Deformation,” *Nature Communications* *16*, no. 1 (2025): 10388, https://doi.org/10.1038/s41467-025-65327-5.

20. X. Wang, Z. Zhang, P. Li, et al., “Ultrastable N‐Type Semiconducting Fiber Organic Electrochemical Transistors for Highly Sensitive Biosensors,” *Advanced Materials* *36*, no. 24 (2024), https://doi.org/10.1002/adma.202400287.

21. H. Jiang, C.-Y. Yang, D. Tu, et al., “Shear-Enhanced Liquid-Crystal Spinning of Conjugated Polymer Fibers,” *National Science Review* *12*, no. 10 (2025), https://doi.org/10.1093/nsr/nwaf331.

22. X. Wang, H. Jiang, H. Wang, et al., “Shear‐Intensified Hybridization of Conjugated Polymer Fibers for Organic Electrochemical Transistors,” *Advanced Functional Materials* *36*, no. 14 (2026), https://doi.org/10.1002/adfm.202515197.

23. S. Ma, J. Wang, W. Wu, et al., “Sequential Cyanation of Polythiophenes: Tuning Charge Carrier Polarity in Organic Electrochemical Transistors,” *Advanced Electronic Materials* *9*, no. 11 (2023), https://doi.org/10.1002/aelm.202300207.

24. W. Yang, S. Ma, S. Gámez‐Valenzuela, et al., “Backbone Tailoring Enables High‐Performance and Stable N‐Type Organic Mixed Ionic‐Electronic Conductors for Synaptic Simulation and Biosensor,” *Advanced Materials* *38*, no. 3 (2026), https://doi.org/10.1002/adma.202512070.
